# The New York Institute of Stomatology

**Published:** 1896-05

**Authors:** 

**Affiliations:** The New York Institute of Stomatology


### Reports of Society Meetings.

### THE NEW YORK INSTITUTE OF STOMATOLOGY.

A MEETING of the Institute was held on Tuesday evening, March 3, 1896, at the house of Dr. C. O. Kimball, 27 West Thirty-eighth Street, the Vice-President, Dr. C. D. Cook, presiding.

The minutes of the last meeting were read and approved.

*The Chairman.*—We are able to give a few minutes to communications on theory and practice. Has Dr. Allan something to say?

*Dr. Geo. S. Allan.*—Some time ago, when I was in Boston, my friend, Dr. E. H. Smith, showed me a series of plates that were designed by his father-in-law, Dr. Shaw, for spreading the upper and lower jaw in regulating. I have tried them in my own practice, and they have been so successful that I begged Dr. Smith to send me on a complete set of the plates so that I might show them this evening. The plates really explain themselves, one being for the purpose of spreading the anterior part of the upper jaw only, as in the ordinary V-shaped mouth, while another one will spread the entire arch. The plates are operated by various little screws, and the force is a positive one; the process of widening the jaw goes on without special inconvenience to the patient, and without the slightest risk of a set-back or loss of time. The amount of pressure can be regulated with the utmost nicety. The same principle is applied to the lower jaw, except that with the lower plates he uses a spring which is detached from the plate. These plates were referred to and partially described by Dr. Shaw in the *Dental Cosmos* for April, 1888.

The principle, I am convinced, is one that can be applied with success in almost any case where spreading of the jaw is required, and in my experience is vastly superior to the Coffin split plate, which I have used a good deal in the past.

*Dr. C. A. Woodward.*—Does Dr. Allan think the principle upon which these plates are based superior to the Jackson method?

*Dr. Allan.*—I do not think the Jackson method is equal to this. The fulcrum, the base of support, is better, and the pressure can be better regulated.

I have another little matter to which I wish to call attention

After a gutta-percha filling is placed in position it is often a matter of a good deal of difficulty to trim off the portions which overlap the margins of the cavity. The ordinary way is to take a warm instrument and cut it off. But that has its difficulties, and it is not a practical way at all times. I have been in the habit for some time of using little knives for that purpose. These knives fit into a socket-handle, and one can go around the neck of the tooth with them and reach almost any position to trim off the superfluous material in much less time than it would take with a warm instrument, leaving also a clean-cut surface. These four sickle-shaped knives constitute the set.

*Dr. C. O. Kimball.*—I would like to call attention to a plan for the registration of operations upon cards, somewhat similar to the card catalogue of a library.

Each patient has a card about nine by seven inches, with diagram of the mouth and numbered spaces for dates, a column of spaces being reserved for records of "cleansing." The operation is noted on the diagram, a single figure referring to the date.

The cards, arranged alphabetically, are kept in the drawer of a case made for the purpose, which is fixed to the wall close to the operating-chair. When work is begun for a patient, this card is taken from the drawer and placed, also in alphabetical arrangement, in a small desk at the top of the case, and kept there till the series of appointments is completed. When each patient comes into the room his card is turned down so that a complete record of all my operations for him lies before me while at work.

Operations on the temporary teeth are noted on a paper diagram temporarily attached to the card, which can be removed and destroyed when the temporary teeth have all been replaced by the permanent ones.

I have had this system in practical use for over twenty-five years, and have proved its value in many ways.

*Dr. W. St. George Elliott.*—I would like to ask Dr. Kimball whether there are not some objections to the card plan of keeping professional accounts. Several years ago I adopted a similar plan, my cards being larger, however, and the diagram of the teeth was on two horizontal lines. The rest of the card was arranged on a plan I had used a number of years, the main feature of which was a three-inch line for remarks for each operation. While practising in London I kept very full accounts of each operation, nature of the case, kind of filling material, kind of mallet, etc. I had expected to draw up some sort of a table, showing the advantages of certain modes of treatment, etc., but retired from practice before I had made any use of the eleven years data. When I re-entered practice, over a year ago, I adopted the card system as an experiment, but was not altogether satisfied with it. The main objections are cost, cumbersomeness, and liability to misplacement; they are not so well adapted to the profession as the usual ledger. I will admit, however, that they have some marked advantages, the principal being, having a card before one instead of a large book, and the ability to withdraw from the lot of cards those of patients who have died or have gone away, a very material advantage.

*Dr. Kimball.*—The system is very simple. It is merely the record of the work, the accounts being kept in another place. The cards are placed alphabetically in a drawer, and kept there. A card is taken out from the drawer and put in the small desk when the patient first comes, and is returned when his work is completed. I have never adopted the keeping of records as fully as Dr. Elliott has stated. My record shows the general kind of operation, what the filling was, when it was put in, and its shape in relation to other fillings, principally for the sake of identifying the work, watching my work, and knowing how it stands. If there are any details which I wish especially to note, as any peculiarity in the filling or operation, I do so upon the margin or back of card. I will exhibit the cards.

*Dr. Elliott.*—In starting a new society, we are naturally ambitious to take a higher position than most societies, and I do not know of any way that would tend to so much usefulness or draw so much attention to our work as to have our papers and remarks properly illustrated. An outline drawing is quite sufficient and is not expensive.

*Dr. E. A. Bogue.*—Something that one of my patients said the other day interested me so much that I would like to repeat it. It is this: "That phosphate filling that was put in for me the other day—after being stearinized or paraffinized—felt smooth just like gold, and had none of the acid taste that such fillings have for a day or two. It is the first time a phosphate filling has ever failed to give me annoyance, although I have had a good many put in my mouth."

*The Chairman.*—I would like to ask if that bad taste occurs when the phosphate has been properly ground.

*Dr. Bogue.*—This lady had had phosphate fillings put in anywhere and everywhere. I do not know how they were done.

*The Chairman.*—I will now call on Dr. J. Morgan Howe, the chairman of the Committee on Materia Medica and Therapeutics, to make a report for his committee.

(For Dr. Howe's paper, see page 286.)

*The Chairman.*—The secretary will now read a communication which has been received from Dr. Louis Jack, of Philadelphia, upon this subject, after which discussion will be in order.

*Dr. Jack.*—In the use of formalin I have not had much experience. Since the range of employment does not appear to be wide, and as it is a powerful agent, one should be cautious in its application.

Its chief value depends upon its rapid diffusibility, and for this reason it is serviceable for the disinfection of the dentine in canals which have been long subjected to the presence of putrescent material. For this condition I have used five-per-cent. solutions with apparent advantage. This would be indicated by my experience to be as strong a solution as it is safe to employ. At this strength it is necessary to protect the adjacent tissues by sealing the saturated pledget in the pulp-chamber and canal, allowing it to remain for a few hours. It is important not to force the solution through the apical foramen: concerning this accident I had a serious experience when, notwithstanding the existence of a fistula, great pain and continued irritation was excited which slowly yielded to treatment. The effect upon the fistulous tract in this case was not curative since the suppuration was not arrested, as would probably have followed the application to the same *situ* of zinc chloride ten grains to one ounce of water.

In exposures of the pulp, preparatory to conservative treatment, I have frequently used and have found a solution of five per cent. of formalin serviceable where there had long been caries with rather broad exposure. For recent exposures, when the bacterial invasion of the dentine were less, I used two and one-half per cent.

As the value of formaldehyde depends upon its quick diffusibility, therefore its use would appear to be confined to conditions which require penetration of structures. Its irritating properties, on the other hand, limit its use as a topical agent for superficial disturbances, as they occur in the mouth. I am not prepared to give an opinion of percentages lower than the smallest rate I have above stated.

*Dr. Allan.*—A little over a year ago I began to use formalin, having heard of it through Dr. Jack, as Dr. Howe did; and my experience with it has been such that I do not well see how I

could get along without it. In the class of cases Dr. Howe describes there is a great deal of irritation and pain, not accompanied by a formation or discharge of pus, but simply due to some peculiar condition, and a permanent stopping is not possible, as intense pain follows closing the root-canal. In these cases I have found that an application of a three-per-cent. or five-per-cent. solution of formalin will almost always work a thorough cure, one application often being sufficient. I treated a lower bicuspid for my brother for nearly a year, possibly longer, and as he lives at Newburgh, I could see him only occasionally. Sometimes I thought I had made a success of it, but he would write me in a day or two that the filling had to be removed. The last time he was down, about two months ago, I treated the tooth with a five-per-cent. solution of formalin, following it with hydrogen dioxide. In a week or so he wrote me that he had some pain after the treatment, but not sufficient to induce him to have the filling taken out. After a little the pain entirely subsided, and the tooth is now ready to fill permanently. While in formalin we have almost an ideal preparation for the class of cases which Dr. Howe has alluded to, we have not yet secured all we need. Formalin will unite with the albuminoids and organic tissues to form a fairly permanent compound; not wholly permanent, for with the access of germs sooner or later putrefaction will take place. There is no doubt of that.

Formalin is employed in the laboratory for hardening tissues preparatory to section-cutting, and is a most valuable agent; but if a mass of tissue so hardened is taken from the solution and exposed to the air of the room, germicidal action soon commences and decomposition and destruction of the tissue follows.

Formalin has an irritant action, irritating the skin or mucous membrane to which it is applied; it has an escharotic action, and kills tissue in strong solutions, but it is not toxic in character, nor is it poisonous when administered even in strong doses; therefore, in weak solutions, it is exceedingly valuable as a mouth wash, and in all preparations to be used on the teeth. I have used formalin a great deal in ordinary cavities where I wish to bring about an aseptic condition preparatory to filling. Formalin is exceedingly penetrating; being an aqueous solution, it is more penetrating than any of the essential oils. I have used it in ordinary cavities, as strong as a ten-per-cent. solution, and what pain followed passed away rapidly and no harm resulted. I am very favorably impressed with its use in such cases. I have not used it long enough to say anything more, except that it is probably the best all-around antiseptic and germicide that we have had brought to our attention. I use it constantly to dip instruments in when cleansing the teeth, removing tartar, etc., about one-half of one-per-cent. solution, not stronger than that, and I have never had a patient complain of pain, irritation, or troublesome action on its part. I am very glad that Dr. Howe has thought best to bring this agent before us, and I heartily indorse all he has said in its favor.

*Dr. S. E. Davenport.*—I should like first to compliment Dr. Howe upon his complete report of this comparatively new remedy. I think it proves that Dr. Allan, the chairman of the Executive Committee, who I think suggested the formation of these committees in our Institute, made no mistake, and that our president, Dr. Lord, knew what he was about when he appointed the chairman of the Committee on Materia Medica and Therapeutics. Through Dr. Howe's courtesy I have been in possession of a vial of formaldehyde, five-per-cent. solution, for six months, and while I am not one who takes readily to new remedies, and use probably fewer medicines in my office than most operators, I have in quite a number of cases which refused to yield to the remedies I have been more accustomed to use, given formalin quite a thorough test. One case I remember in particular,—I will not enter into details, except to say of it that twice within three weeks did I leave my dinner-table to remove the temporary stopping from this inferior third molar, it having been treated with other remedies,—with electrozone once, by the way,—but when formalin was used as a dressing I was able to eat my dinner in peace, and the tooth is now filled and comfortable. I think it might be well, if Dr. Howe is willing, not only for our information, but for the readers of our proceedings, for him to say a few words regarding the reduction of formaldehyde from its forty-per-cent. solution to the different strengths which have been mentioned in his report.

*Dr. Howe.*—I think the easiest way that it can be presented is to regard the forty-per-cent. solution as forty parts of the drug formaldehyde, divided by a unit of water, instead of a hundred parts of water, which it really is. If we take an ounce, or a teaspoonful, or any amount, and consider it as stated, and desiring a one-per-cent. solution, we add thirty-nine teaspoonfuls of water,—supposing that we use a teaspoon to make our dilution,—and we have divided the forty parts of formaldehyde by forty parts of water instead of one, which we took as our starting-point. If we want a five-per-cent. solution, we add seven teaspoonfuls of water to one teaspoonful of the forty-per-cent. solution: we have divided the forty parts of the pure article by eight instead of one, and have obtained a five-per-cent. solution.

*Dr. Bogue.*—Will Dr. Howe tell us whether this solution is a permanent one, or subject to deterioration?

*Dr. Howe.*—I think it is very little subject to deterioration. One writer only, whose remarks on that point I have observed, claims that it requires to be fresh. On the other hand, there are many who claim that it is remarkably stable. Almost all the alcohols are, I think, liable to full oxidation, which converts them into acids, as is shown in the facility with which wine and cider are converted into vinegar, if there is access of air. But the oxidation of methyl alcohol seems to be capable of permanent restraint at the aldehyde condition. It is claimed that forty-per-cent. solutions, kept in stoppered bottles, showed only a maximum increase of one-tenth per cent. of formic acid in eleven months. I have some that has been in my office over eleven months that seems to be perfectly good.

*Dr. Allan.*—Fearing that I may be misunderstood I will say that the formalin of commerce, which is a forty-per-cent. solution of formaldehyde, has been my basal preparation, and the different solutions to which I have referred have been made by reducing formalin by the addition of water.

*Dr. Davenport.*—Then Dr. Allan's ten-per-cent. solution is Dr. Howe's four-per-cent. solution.

*Dr. Howe.*—If the manufacturers of the solution tell the truth, they have made a forty-per-cent. solution of formaldehyde in water. I had not thought of accepting this as the unit of value, because it is a solution of known strength, and is presented in this form for convenience only. I think the name formalin is used by most persons only as a contraction of the real name, and not as adopting the production of one manufacturer as the original article with which we are dealing. I should not consider it desirable to adopt the proprietary name of an article in which no one has any proprietary rights, especially as by so doing we would be liable to lose sight of the chemical nature of the article. And I think it would lead to confusion to regard a solution as if it were the original substance, and refer to percentage solutions of a solution, instead of referring back to the compound itself.

*Dr. C. F. Ives.*—It is a little less than a year since Dr. Howe gave me a sample of formalin, and I think that it is one of the best remedies I have had. I want to speak of a case which came to me a few weeks ago. It was an inferior first molar. A dentist out of the city had treated it for three or four months. It was so irritable that every attempt to close it resulted in pain, which made necessary the removal of the stopping within from one to three hours, and, becoming discouraged, the dentist concluded to let it alone. The tooth finally became so troublesome that the lady could not pack in cotton sufficiently tight to exclude food without having to remove it in the course of an hour. That is the condition it was in when she came to me. I applied the dam and dried out the cavity thoroughly, finding nothing in the roots to indicate any particular trouble. After a little necessary cleansing, I applied a five-per-cent. solution of formalin, carrying it to the extremity of the roots, and sealed with gutta-percha. I did not see the patient again for ten days, and when she came my first question was about the tooth. She said, "I have forgotten all about it, I have had no trouble from it since." I immediately filled both roots, and put a permanent filling in the cavity. That was two months ago, and I have heard nothing from it since. The day before yesterday a lower bicuspid having a very long and tortuous canal presented itself. In this case the pulp had been destroyed in Syracuse, and the lady was directed to attend to it when she reached here. She neglected it for four or five months, and when I saw her the face was very much swollen and she was suffering severely. I introduced a fine broach and passed it to the end of the canal, and, finding no opening, went through with a drill made from a fine banjo string, and applied a two-and-a-half-per-cent. solution of formalin. The result was that the pain ceased. Then I made one more application, and it is ready to fill permanently. It seems that just in those cases which resist everything else, formalin has a remarkable effect. I have made it a practice to swab out all cavities before filling with a five-per-cent. solution, and it has a marvellous action.

*Dr. Z. T. Sailer.*—I take it for granted that the medicine acts beyond the root, because, if the root is cleansed thoroughly and filled to the apex, trouble follows just the same as if a lesion exists in the membrane outside. It is a very great irritant, and I cannot see how it becomes curative, although it is possible, according to the evidence to-night. Dr. Allan cites the case of his brother, who, being a dentist, should be able to give us positive information in regard to this important question.

*Dr. Howe.*—I suppose that the trouble in the class of cases referred to, for which this remedy seems to be especially adapted, is a condition of irritability beyond the apex of the root; not *in* the tooth. The fact that formalin is a great irritant, and at the same time capable, in dilution, of allaying irritation, is a peculiarity of the remedy that pertains to other drugs also, but generally in less degree. Medicinal action is something that no one, I think, can explain. I do not know that there is any explanation of the way each drug has its peculiar medicinal or toxic effect. Each one has an individuality in its effects, and the fact that it does so is something that we are glad to act upon, when we have the knowledge; accepting the fact and doing the best we can with it.

*Dr. Allan.*—The drug formaldehyde in strong solutions is a direct irritant, destroying the life of the part to which it is applied. In weaker solutions, not strong enough to exert a necrotic action, but having decided germicidal properties, it quiets pain by destroying the germs of putrefaction and inflammation. The pain from using a ten-per-cent. solution of formalin even on a sensitive tooth is very transient. In repeated applications I have never known the pain to last over five or ten minutes. This is, as I understand it, an explanation of the apparently contradictory properties of the drug.

*Dr. Howe.*—It is to be noted that germicidal properties alone do not fulfil our requirements, because no other germicide is capable of accomplishing what formalin does in certain conditions. Peroxide of hydrogen, bichloride of mercury, and electrozone are very effective in that sphere of action, but neither of them will reduce the peculiar irritable condition we have considered in the tissues just beyond the apex of a pulpless root. Formaldehyde does it very promptly. Its effect is different entirely from germicidal action, or is an effect in addition to that of germicidal action.

*Dr. A. J. Weed.*—I do not believe there is much irritative effect. I have used formalin to attack an abscess, washing out with a little salt-water and packing with antiseptic cotton dipped in formalin. My object was to see if there was periosteal inflammation. The patient did not complain of any decided pain.

*Dr. Howe.*—It did not produce pain?

*Dr. Weed.*—She did not complain of any more pain than from packing with cotton. I used a five-per-cent. solution, simply wetting the end of the cotton.

*Dr. Howe.*—It is in just such cases that formalin is so useful.

*Dr. Kimball.*—I am glad your attention has been called to the medical aspect of this question, because I think we have sometimes overlooked that in our treatment of the peculiar class of cases that has been described. In newly-opened pulpless teeth I have used for many years a first dressing of tincture of aconite, bearing in mind its therapeutic action on the adjacent tissues. The result has been good, producing almost no irritating effect, and allaying any tendency to inflammation which may have arisen from admitting air to the long-closed cavity. Its effect has been very satisfactory, but I am glad to have this additional anchor to use in these troublesome cases.

*Dr. Bogue.*—I want to ask for some help, but before asking for it I should like to make a little acknowledgment to Dr. Howe for his kindness in giving me a sample of formaldehyde some months ago. Unfortunately I left it behind me last summer, and could not find it until two or three weeks since. I have had occasion to use it once or twice, each time with marked success. I found myself very ignorant in the use of it, and wanted to know more about it, and my presence this evening has been fortunate for me. The help that I want is right in the line of this committee's report, and it is also in the line of operative dentistry. We have heard a good deal of late about cataphoresis; we have been deluged with advertisements from men, professional and otherwise, in regard to an instrument called a volt-selector, and many of us have invested our pennies in it, and some of us, perhaps, have run in debt for it. I suppose we must have some new plaything every little while, or we are not happy. I should like to have the members of this society tell me what is the use of the so-called "volt-selector." It is said to produce certain effects in two directions where we need assistance very much, and possibly in a third. Those two directions are in the anæsthetization of sensitive cavities which we have to excavate, and the driving electrically of a bleaching agent into the dentine for the purpose of bleaching discolored teeth. Those who have had more experience with cataphoric processes than I have will probably be able to send to the committee, of which your humble servant is chairman, and which wants to make a report, some positive information upon these points. I received a telegram a few moments since from Dr. Palmer, who requests me to say in regard to this matter that he had several patients—perhaps five or six—for whom he had used cataphoresis with cocaine to obtund the sensibility of cavities which he was excavating, and in almost every instance—I say almost so as to be on the safe side—the patient complained of subsequent pain. And in one case where I used it myself the patient said, "Well, I do not want that thing any more; as a rule, when I go away from here with a tooth filled that is the last of it, but I have been nearly thirty-six hours now suffering from that tooth which was filled, and I do not like that." If that experience has been had by others I should be very pleased to know of it. I think we ought to give a fair, just, and impartial examination into the question. I think that is what we are doing in this society; and if any members can give the result of their knowledge in that direction I shall be obliged.

*Dr. Davenport.*—I might call Dr. Bogue's attention to a short article in the March number of THE INTERNATIONAL DENTAL JOURNAL, from the pen of Dr. Rollins, of Boston, on this subject; and judging from the little Dr. Rollins wrote, I think it might be worth Dr. Bogue's while to ask Dr. Rollins to say more upon the same subject.

*Dr. Bogue.*—Very much obliged. I will do so.

Adjourned.

S. E. DAVENPORT, D.D.S., M.D.S.,
*Editor The New York Institute of Stomatology.*

